# Consciousness as an Emergent Phenomenon: A Tale of Different Levels of Description

**DOI:** 10.3390/e22090921

**Published:** 2020-08-22

**Authors:** Ramón Guevara, Diego M. Mateos, José Luis Pérez Velázquez

**Affiliations:** 1 Integrative Neuroscience and Cognition Centre (INCC UMR8002), University of Paris and CNRS, 75270 Paris, France; 2 Department of Physics and Astronomy, University of Padova, 35131 Padova, Italy; 3 Department of Science and Technology, Universidad Autónoma de Entre Ríos, Paraná 3100, Argentina; mateosdiego@gmail.com; 4 Instituto de Matemática Aplicada del Litoral (IMAL-CONICET-UNL), Santa Fe 3000, Argentina; 5 The Ronin Institute, Montclair, NJ 07043, USA; jose.velazquez@ronininstitute.org

**Keywords:** neural synchronization, consciousness, quantum biology, brain dynamics, brain connectivity

## Abstract

One of the biggest queries in cognitive sciences is the emergence of consciousness from matter. Modern neurobiological theories of consciousness propose that conscious experience is the result of interactions between large-scale neuronal networks in the brain, traditionally described within the realm of classical physics. Here, we propose a generalized connectionist framework in which the emergence of “conscious networks” is not exclusive of large brain areas, but can be identified in subcellular networks exhibiting nontrivial quantum phenomena. The essential feature of such networks is the existence of strong correlations in the system (classical or quantum coherence) and the presence of an optimal point at which the system’s complexity and energy dissipation are maximized, whereas free-energy is minimized. This is expressed either by maximization of the information content in large scale functional networks or by achieving optimal efficiency through the quantum Goldilock effect.

## 1. Introduction

The relation between behavior and brain function is the main task of cognitive neuroscience. In particular, one of the biggest queries in cognitive sciences and philosophy is the emergence of consciousness from matter [1,2]. A natural question to ask is at what level is consciousness generated, that is, what is the neural substrate of conscious experience and where it is located. Different possibilities have been explored, ranging from the cellular level to the level of the whole or at least large parts of the brain [3].

One reason that makes this question hard to answer is that, as with other primordial questions in science, there is not a precise definition of consciousness, one that is accepted by all the scientific community [4] (but see some proposals about the advantages of a less strict definition of consciousness in [5]). Similar conundrums can be found all across biological sciences. Even the concept of life itself leads to several possible definitions and diverse proposals to define life had been advanced during the years, more or less emphasizing particular attributes of biological systems [6]. Furthermore, if one were asked where in an organism resides a property called “life”, in other words, what part of the organism is alive, one would probably consider the question misleading or simply wrong. One would eventually conclude that life is a global property that has no specific scale, and, in fact, considering that basic aspects of life are exchange of energy, reproduction and compartmentalization, inorganic compounds which have these properties could be considered “alive” to some extent [7], as similar principles of organization apply to the living and the nonliving [8]. The organism is a complex system at all possible scales, from systems made up of organs down to the molecular scale, and each part contributes to the whole in an irreducible way. Failure at any scale can lead to the collapse of the organism; consider, for example, damage to the DNA structure by ionizing radiation at the molecular level, or heart failure at the organ level.

Where in the nervous system (or more generally, in the organism) consciousness resides may constitute a question of a similar nature. One possible answer could be that consciousness does not reside within any specific structure and that it cannot be clearly associated with a particular spatial scale. Another possibility is that consciousness appears at a specific scale, associated with a structure or group of structures. This is the question we address here. We start by acknowledging the overwhelming evidence supporting the idea that integration in large-scale neural networks may constitute the underlying mechanism of conscious phenomena, at least at the mesoscopic or macroscopic level of description.

However, we also address the possibility that conscious phenomena can result from, or at least be enhanced by, non-trivial quantum effects. Does quantum physics plays a role in the emergence of conscious phenomena? Undeniably, quantum mechanics plays a fundamental role in chemistry and therefore in physiology. Some authors have argued that it may also have an indirect but important role in neurochemical processes related to cognition [9]. However, the idea that quantum effects could play a direct role on consciousness remains speculative [10,11]. Nevertheless, alternative explanations to prevalent paradigms do exist. In particular, several authors have proposed during the course of the last few decades, the possibility that consciousness could arise as a result of non-trivial quantum mechanical processes, such as quantum coherence and entanglement (for a magnificent recent review see in [12]). Here, we find common principles underlying both large-scale and quantum theories of theories of consciousness, by focusing on a connectionist approach to the emergence of consciousness. We propose the hypothesis that consciousness is a multiscale phenomenon that could appear both as a result of large-scale interactions in neural networks or due to non-trivial quantum phenomena within cells.

## 2. Connectionists Theories of Consciousness

Several theories of consciousness are strongly based on a connectionist approach, that is, on the idea that large webs of interacting neurons are fundamental to understand brain functionality, cognition, and behavior [13,14]. These are “global” theories of consciousness, in that typically large brain areas are activated and necessary for the emergency of consciousness [15,16]. These theories provide a point of view according to which it is possible to properly characterize conscious phenomenon in terms of certain properties common to all of them, and they have offered means to quantify them by identifying concomitant brain processes and possible underlying mechanisms. In the words of the authors of two influential neurobiological theories of consciousness, the dynamic core hypothesis [2,17] and the Integrated Information Theory (IIT) [18,19], “instead of arguing whether a particular brain area or group of neurons contributes to consciousness or not, our strategy is to characterize the kinds of neural processes that might account for key properties of conscious experience” [17].

For example, the Baars’ global workspace theory [20] has used the traditional Cartesian argument of consciousness, an influential idea in cognitive sciences, in association with specific neural hypothesis based on electrophysiological evidence [21]. In the Cartesian theater metaphor, consciousness is an illuminated part of a stage representing immediate memory. This spotlight is shifted across the stage, directed by attention under executive action, whereas the rest of the theater is in darkness and unconscious. The bright spot correlates with the activation of a specific sensory projection cortical area (e.g., visual, auditory), whereas other cortical areas are simultaneously inhibited as they correspond to non-illuminated parts of the theater. Contents of conscious states are then sent to specialized networks all around the brain, corresponding to the audience sitting in darkness; this may happen in the brain through corticocortical or corticotalamic pathways. Specifically, the spotlight region content is chosen by attentional mechanisms, using bottom-up limbic and thalamic pathways or from executive top-down prefrontal cortex input [22]. Therefore, the most important role of consciousness is that of integrating and coordinating a large number of specialized networks that would otherwise operate autonomously [23].

Similarly, in the implementation of the global workspace theory due to Dehaene, Changeux, and colleagues, a pattern of global activity is generated in the workspace when sensory stimuli activate excitatory neurons with long-range corticocortical axons [24]. Such patterns inhibit other possible patterns of activity that therefore remain unconscious, preventing the processing of alternative stimuli.

According to some of the “global” theories of consciousness, it is possible to identify two general properties of conscious experiences: integration and differentiation [17,18]. Integration reflects the unity of conscious experiences, in the sense that each such experience is unique, so it cannot be decomposed into independent components. Differentiation refers to the enormous amount of potential conscious experiences that are simultaneous, that is, the extremely large repertoire of possible conscious experiences of which one is selected.

In correspondence with these general properties of conscious phenomena, IIT, the dynamic core hypothesis, and other related theories of consciousness identify neural processes that underlay conscious experience [18]. The first such process is the activation and deactivation of widely distributed brain areas, particularly in the thalamocortical system (segregation). The second is the integration through strong and rapid reentrant interactions between differentiated neuronal groups. In simple words, differentiation is achieved by the simultaneous activation of distinct and widely distributed neuronal groups, and integration refers to the process of connection of these groups through reentrant interactions to achieve a unique conscious experience.

A crucial element in connectionists approaches is that consciousness appears as an emergent property of large neuronal networks, requiring the interaction of neurons to form a web sufficiently complex such as to sustain conscious experiences. In such view, neurons are crucial, that is, necessary but not sufficient to generate consciousness. This is not an exceptional perspective in cognitive neuroscience, but rather representative of contemporary ideas. In the dominant computing argot, neurons constitute the computing units within large networks, and computation is performed at the synaptic level. We can abstract out the crucial point: the interaction (integration) of a large ensemble of processing units (segregation) leads to emergent phenomena, among them consciousness. Thus, conscious experience reflects such underlying substrate expressed in the neurophysiological properties of integration and segregation.

However, what is the mechanism of brain integration underlying conscious phenomena? Temporal correlations are known to play a crucial role in the functioning of the brain, cognition, and conscious experience. Indeed, it has long been recognized that temporal correlations of neural activity are crucial for communication between brain areas and between neurons [25,26,27,28,29].

Temporal correlation in the brain is often associated to the concept of neural synchronization. It is well known that nonlinear coupled autonomous oscillators (self-sustained oscillators) show synchronization phenomena [30]. Synchronized oscillators show temporal correlations, specifically correlations in the phase of the corresponding signals. Neurons are not an exception, and synchronized neuronal activity (phase synchronization) has been found at multiple scales within the nervous system [31], contributing to the so-called “braniweb” [27]. Not only do individual neurons synchronize their activities in local regions, but so do large neuronal networks [32].

It is currently believed that neural synchronization is the mechanism at the basis of integration in the brain [27]. Neural correlations are therefore of crucial importance in connectionist theories of brain activity and cognition, and in particular in connectionist theories of consciousness, such as IIT, because they underlay the property of integration in conscious experience.

Synchronization is also crucial for the development of brain rhythms, that appear to have a huge relevance in cognitive processes, including conscious experience [31]. Indeed, synchronization is required for neurons to produce an activity that is quasi-periodic across large brain areas, such as the rhythms observed in electroencephalographic (EEG) recordings [33]. They appear as a result of feedback activity across large neuronal loops, and they have been associated to a myriad of cognitive functions [34]. Of crucial importance in the current context, the gamma band activity (around 40 Hz) has been associated with high cognitive functions, including consciousness; however, the generation of gamma activity may be a consequence of the natural activity of neurons during conscious experience: gamma frequencies reflect the emergence and dissolution of communication among many neuronal ensembles, fluctuations providing the variability needed in brain dynamics to process information leading to adaptive behaviors [35].

Several studies have emphasized the importance of the thalamocortical system and gamma band synchronization in the generation of conscious experience [36]. In particular, it seems plausible that synchronous oscillations in thalamocortical loops creates conscious states.

It is also important to notice that the breakdown of neural synchronization may lead to pathological brain dynamics. This is exemplified in diseases such as epilepsy and Parkinson disease, characterized by abnormal synchronization of brain activity [37]. In the case of epilepsy, over-synchronization in extended brain areas leads to the large, periodic discharges observed in EEG recordings during seizures and the concomitant loss of responsiveness. Some authors suggest that during epileptic absent seizures, where consciousness is temporarily lost, there is a disruption of the thalamocortical “resonance” and gamma band synchronization which leads to disorders of consciousness [36].

A crucial point in connectionist theories of consciousness is the relationship between consciousness and complexity. According to the dynamic core hypothesis, complexity means that the dynamic core must be highly differentiated. The dynamic core also correlates with the conscious state of the subject [17]. Therefore, it is expected that complexity is higher for “more conscious” states. For example, complexity of deep sleep stages should be lower than complexity during awake states.

**Figure 1:**
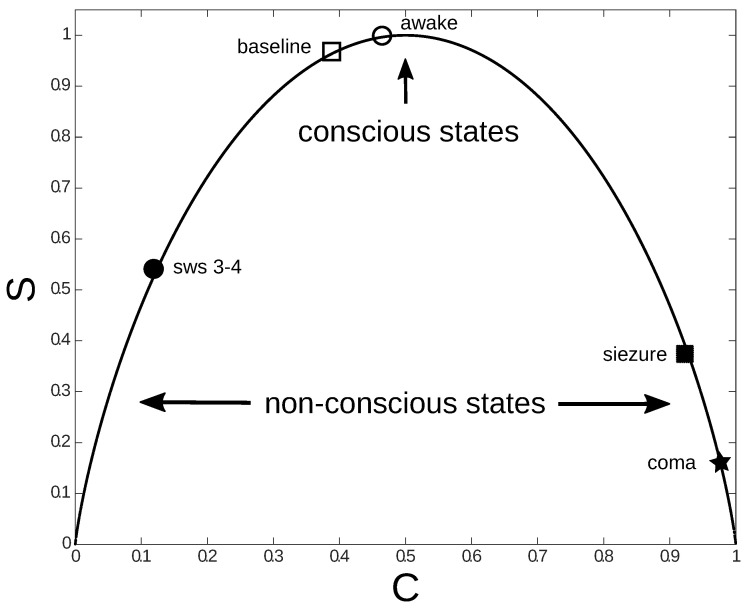
Shannon entropy (S) of brain networks (a measure of network complexity) as a function of network connectivity (a measure of correlation in brain activity) for conscious and non-conscious states. Each data point is a measure of brain activity using either electroencephalography or magnetoencephalography. baseline = brain activity before a seizure. sws 3–4 = slow wave 3–4, a phase of deep sleep. During awake and “baseline” (non-seizure) states (conscious states), entropy is large, as compared with coma, seizure, and sws 3–4 states (non-conscious). Conscious states appear at intermediate values (not too large, nor too low) of correlations in brain networks (C), also corresponding to intermediate values of noise in brain activity. The inverted U curve represents the dependency of S with C, calculated using a statistical mechanics model [38].

Based on a statistical mechanics approach, some features of brain organization optimal for sensory processing and that correlate with consciousness were recently identified [38,39,40]. Specifically, scalp electroencephalograpy (EEG), intracranial electroencephalography (iEEG), and magnetoencephalography (MEG) techniques were used. The results are encapsulated in Figure 1, that requires some clarifications, as we briefly do in the following.

Using the above mentioned techniques, brain activity was captured by sensors that measured the electric or magnetic fields generated by neurons (postsynaptic neuronal currents generate such fields). In [38], the synchronization of EEG/MEG activity was calculated at all possible pairs of sensors, representing a measure of “coupling” between brain areas (the reason is that phase synchronization between nonlinear oscillators is also a measure of the physical coupling between them [30]). For example, consider an EEG recording of brain activity. If time series $x_{j}\left(t\right)$ and $x_{k}\left(t\right)$ are measured at two different electrodes, phase synchronization between these electrodes is given by the so-called mean phase coherence [41], that is, the averaged phase correlation between the time series,

(1) $$R_{jk}=\left|<e^{i\Delta \theta_{jk}}>\right|,$$

where $\Delta \theta_{jk}=\theta_{j}-\theta_{k}$ is the instantaneous phase difference between the signals at the two electrodes. The instantaneous phases θ(t) were extracted from the signals measured at the electrodes by means of the analytic signal approach (this is a standard technique in time series analysis [42]): For each time series x(t), an analytic (complex) signal z(t) is derived as

(2) $$z\left(t\right)=x\left(t\right)+iH\left[x\left(t\right)\right]=Ae^{i\theta },$$

where H[x(t)] is the Hilbert transform of the signal x(t). It can be seen in the expression above that the phase is the angle of the analytic signal. For every pair of electrodes (for each *j* and *k*), this gives a measure of how much the signal at those electrodes are synchronized (*R* values range between 0 (low synchronization) and 1 (high synchronization)). All possible pairs of electrodes were considered, and a connectivity network (matrix $R_{jk}$) was built, with nodes representing individual electrodes and edges representing the amount of phase synchronization between them. This constitutes a “functional connectivity network” (to distinguish it from a purely anatomical brain network). It gives a measure of how much correlation exist between brain regions.

A new measure of complexity, based on information theory, was used in [38] in order to contrast different brain states (such as sleep states (“sw 3–4”) and seizures in Figure 1), by calculating the complexity of the corresponding functional connectivity networks. To this end, connectivity networks were further simplified: a threshold was used to decide if two electrodes were connected or not. After this transformation, all pairs of nodes in the network were considered to be either connected or disconnected, that is, the corresponding brain areas were considered to be either coupled or uncoupled. The amount of connectivity in the network (*C*) is given by the number of connected pairs of electrodes, or equivalently, the number of brain areas functionally coupled (the X-axis in Figure 1). In [38] the complexity of the connectivity network was given by the Shannon entropy of the network (Y-axis in Figure 1), that is,

(3) S=lnΩ,

where Ω is the number of possible network configurations with a given amount of network connectivity (*C*). In other words, the calculated Shannon entropy represents the number of possible networks that can be built with a given amount of global correlations (couplings) among different brain areas, and is therefore a measure of brain’s functional complexity.

The data points in Figure 1 (squares, stars, and circles, black and white), are pairs of (*C*,*S*), calculated from brain recordings for different brain states. In the figure, deep sleep states (“sw 3–4”) and “awake states” of a normal subject are considered, as well as data recorded from an epileptic patient before (“baseline”) and during a seizure (“seizure”), and data from a patient in coma (“coma”). The inverted U curve (continuous curve) is the dependence between *S* and *C*, found theoretically by a standard combinatorial calculation (for more details about this derivation see [38]).

Normal wakeful states (“awake” and “baseline” on the top of the inverted U curve) were characterized by larger number of possible configurations of interactions between brain networks (representing highest Shannon entropy values, that is, the largest complexity), as compared with non-conscious states (“sw 3–4”, “seizure”, and “coma”) . This suggests that the information content is larger in networks associated with conscious awareness and that consciousness could be the result of an optimization of information processing [38,39,40]. These observations also suggest a fine-tuning of parameters in order for brain dynamics to achieve maximal efficiency. Indeed, conscious states are typically near the maximum of the curve of complexity vs connectivity (Figure 1). As connectivity increases with increase synchronization, this implies that networks are more efficient in information processing and consciousness emerges at intermediate values of synchronization: too high or too low values of synchronization were associated to low information content in functional networks, or equivalently, low network complexity. Indeed, low values of complexity were observed in deep sleep stages and during generalized epileptic seizures, where consciousness is lost.

Another way to look at this fine-tuning is to describe it in terms of a trade off between noise and phase correlation in the system. Too much noise typically indicates a very low level of correlation, a system that is too random. On the other end of the spectrum, if the system has too little noise and too much correlation (for example, during a seizure) it is excessively ordered and cannot convey information. Therefore, it seems that consciousness appear within a region of brain dynamics where noise and correlation values are intermediate (no too low nor too high). This is in agreement with ideas going back for decades about the emergence of complexity in biological systems, such as the “edge of chaos” concept of Stuart Kaufman [43]. According to these ideas, complexity arises in the border between order (as in a crystalline lattice) and disorder (as in a gas).

Furthermore, it seems natural to treat the complexity of brain interactions, including phase correlations in functional networks and the influence of noise on cognitive states, within a probabilistic framework using thermodynamic methods. Indeed, in the physical sciences this has been the traditional treatment to characterize systems composed of a myriad of interacting parts, whose detailed (microscopic) features are in practice impossible to be obtained but for which thermodynamic (emergent) quantities such as temperature, entropy, and others can be perfectly obtained and constitute a macroscopic description. In a recent article, such a framework was developed and applied to the problem of the emergence of consciousness [35]. The authors emphasized the fact that neuronal networks are out of equilibrium systems where dissipative structures can be created. They also discuss the important role of free energy minimization and its connection with energy gradients in the system. They study the question of the efficient dissipation of energy by brain tissue, also in connection with the existence of pathological states such as in epileptic seizures and coma. They argue that in such states, fluctuations in synchronization are small, and correspondingly there is less dissipation of energy, as compared with normal brain states. Taking into account the results obtained in [38] and described above, that means that during functional and conscious states, dissipation is large. Concomitantly, a large number of metastable states are created (a notion that is related to accounts of the nervous system in terms of criticality, see in [44]), which increases the adaptability of the organism by providing a very flexible repertoire of transient dynamical states. This is in line with previous accounts on the role of dissipation during pattern formation in the context of nonequilibrium thermodynamics [45]. It is also in line with the free-energy principle of Friston [46], which attempts to unify action, perception, and learning within a unique probabilistic framework. According to this principle, any self-organized system in equilibrium with its environment tends to minimize its free-energy [47]. For example, biological organisms tend to maintain homeostasis, and to do so they minimize disorder and free energy. As for the sensory system, organisms tend to minimize surprise (recall that entropy is the average of surprise), or in the words of Friston “biological agents must avoid surprises to ensure that their states remain within physiological bounds” [46]. They do so by minimizing free-energy, that is an upper bound of surprise. Free-energy can be manipulated by organisms by either changing their sensory input or their “internal representations”, that is, either by acting on the world or by changing their representation of it.

Summarizing, we identify four properties that are common for the emergence of consciousness in a global connectionist approach:

1. The existence of functional networks that are activated and deactivated in accordance with concomitant conscious phenomena. Such networks are global because they expand across large areas of the brain.
2. The presence of temporal correlations (neural synchronization) within networks relevant to consciousness.
3. A fine-tuning of network parameters is associated with the emergence of consciousness. Consciousness appears at intermediate levels of noise and network correlation. The complexity of the networks is close to maximal for conscious states and decreases on altered states of consciousness.
4. Such fine-tuning is driven by minimization of free-energy and maximization of energy dissipation.

## 3. Towards a Multiple-Scale Theory of Consciousness

When characterizing the underlying cognitive phenomena of networks, the identification of the constituting units of the network is fundamental. According to the influential ideas put forward by global connectionist theories of consciousness, such entities are restricted to either individual neurons or neuronal networks. However, extremely complex networks of interacting units do exist within cells and, in particular, within neurons. Could it be that a connectionist approach to consciousness can be also established at the infra-neuronal level, with subcellular mechanisms contributing to the emergence of consciousness? Interestingly, brain activity at the molecular level is very rich, and networks of all sorts can be identified within cells (consider, for example, metabolic networks).

More generally, a purely connectionist approach leads naturally to the following question. Why consciousness does not emerge from any network (not necessarily neuronal) fulfilling the necessary ingredients (integration and differentiation) for its emergence? Complex networks are widespread in biological systems, and they can be identified at multiples spatio-temporal scales. Consider an extreme case: is a sufficiently complex ecological network conscious? This is a very difficult question, that is beyond the scope of our text. Instead, we focus on networks of interactions within the nervous system. We have to consider that the nervous system has evolved to process information and to organize the responses of organisms to environmental changes in a timely fashion, contrary to other networks that are not specific in this sense. As conscious experiences, at least at the psychological level, are intimately related to perception and cognition, it makes sense to analyze networks within the brain or within neurons.

An important property of the nervous system is that its architecture is extremely complex and there is an intricate connection between structures at different scales. For example, the detection of a few photons at a single retinal neuron may produce a cascade of neural processes leading to the perception of light. In humans and other mammals, it is argued that the first steps of this cascade are actually unconscious, and only when large brain areas are activated this percept effectively enters into the realm of conscious awareness. In much simpler animals with a very small nervous system, the cascade is finished much sooner. Some could argue that such animals are not conscious. However, several authors have suggested that consciousness may be a general property of vertebrates, including very early forms [48].

Of course these perspectives depend on the definition of consciousness one adopts, but we have to admit there is no single definition that satisfies all, and we will not delve into this point here, rather only will mention that, as commented above, there are advantages if less strict definitions of consciousness are adopted [5]. To wit, just like the notion of life, consciousness can be defined by enumerating characteristics: features like self-reproduction, compartmentalization, etc. can be used to “define” life and consciousness can be described by sensorimotor actions, self-awareness, memory, etc. In this manner, “primitive” creatures and even microorganisms can be endowed with some sort of primordial consciousness, as these entities possess some features of consciousness like response to environmental changes, memory, and others. However, few numbers of neurons and networks in worms do not allow them, presumably, to display self-awareness—even though we note that worms have a withdrawal reflex in that these animals respond to external cutaneous touch but not to self-induced touch so they seem to distinguish between inputs from the outside world and the self-induced sensory input, phenomenon that occurs because of the corollary discharge: the transmission of a copy of motor commands to sensory areas where the expected sensation is generated [49]. In the final analysis, these behaviors represent a continuum in cognition, the essentials being present in primordial nervous systems, and it should always be kept in mind that cognition and consciousness developed in a graded, continuous fashion, these are not static properties but a dynamic processes [50].

In any case, complex structure and dynamics have been identified at many levels within the nervous system. One important property of complex systems and processes is the apparent lack of a specific, dominant scale, a property that also pervades brain architecture and function [51]. Indeed, scale-free properties are common in neuronal networks. Just to mention an example, the electric signal detected in electroencephalographic recording exhibits scale free-dynamics, characterized by the so-called 1/f property of the power spectrum [52]. It is then arguable that phenomena at the cellular level may have a very important impact on conscious experience, an assertion that may be speculative and controversial and yet not ruled out by experimental evidence.

Thus, what criteria do we use in order to identify networks correlates of consciousness or self-awareness, apart from those already known—large neuronal networks in large brains—that constitute the central theme of modern neuroscience? Above, we proposed that we can follow a connectionist approach, and identified three properties that such networks should satisfy. We also propose that a fundamental property of such networks is the existence of synchronization phenomena, or resonance phenomena leading to important levels of temporal correlation in the network. We emphasize that synchronization is a widespread phenomenon in nonlinear dynamics, and therefore is commonplace among complex interconnected networks, with abundant examples in physics, chemistry, and biology. Mathematically speaking, synchronization is the adjustment of rhythms of weakly nonlinear oscillators [30]. However, “neural synchronization”, as understood in neuroscience, is an umbrella term compressing all sorts of phenomena in the nervous system where temporal correlations are present [53], for example, there may be resonant phenomena in neurons but also coherency of the mean field of large neuronal networks. We propose that is neural synchronization—both in a strict mathematical sense and in the neuroscientific broad sense—that underlies conscious phenomena, because it leads to temporal coordination and correlations within neuronal networks, a crucial ingredient for conscious phenomena, that is, integration of information.

The question is then if temporal coordinated activity has been identified in networks within cells, and to verify that such networks correlate to conscious phenomena. Curiously, an interesting and unexpected candidate has recently appeared in approaches to the problem of consciousness, based on quantum physics rather than classical physics, and associated to subcellular or even molecular structures. We refer here to this approach as “quantum consciousness”. Temporal coordination in that case comes in the form of quantum coherence and entanglement. We will dedicate the rest of the article to articulate this idea.

## 4. Quantum Effects in Biology

Is there any relevance of quantum physics for living organisms? One possible answer is that ultimately any kind of matter is described by quantum mechanics. Indeed, there is one obvious way in which quantum mechanics enters the realm of biological phenomena: at the biochemical level, molecular structure and interactions are affected by quantum phenomena, because quantum effects are important at the scale of atoms and molecules. This has important consequences, for example, in the enzymatic reactions occurring in biological membranes within cells. However, we do not refer here to this kind of quantum phenomena, which is rather trivial, in the sense that they can be equally described by approximations using classical physics. We refer, instead, to irreducible quantum phenomena that require in one way or another superposition of quantum states, typically at a larger scale, compared to the molecular scale, and involving large polymers and complex chemical reactions. Nontrivial quantum effects are related to quantum coherence. Coherence appears because in quantum mechanics particles have wave-like properties, in the sense that their probability distributions are given by a wave-like equation (Schrödinger equation) [54], and therefore, as is typical for other waves, are subject to interference phenomena. A very well-known example is Young’s double-slit experiment. Individual particles pass through each of the slits, but the ensemble is described by the probability distribution of the particles, which has wave-like properties including interference. Under certain conditions, quantum coherence can appear at the macroscopic scale, as in Bose–Einstein condensates. The destruction of coherence by interaction with the environment is called decoherence. Although more difficult to prove in a biological context, some authors also refer to the possible physiological effect of a purely quantum correlation, called entanglement [55], impossible to describe in terms of classical physics [56]. Entanglement is a phenomenon that results when the wavefunction describing the state of a group of particles cannot be factored out as the product of the sates of individual particles. Entanglement is a simple consequence of the superposition principle applied to multiparticle systems. It implies that the properties of certain configurations of particles are intrinsically intermingled (entangled) in such a way that results of experiments on different particles are correlated in a nontrivial way, impossible to explain in terms of classical physics Finally, quantum tunneling, another quantum effect in which particles have non-zero probability of passing through a barrier potential, has also been associated with biological phenomena such as olfaction and neuroreception [12].

Of great importance for the argument we develop here, quantum coherence and entanglement lead to temporal correlation between particles, in the same manner as synchronization lead to correlation between nonlinear oscillators. This is the crucial analogy we emphasize in this article. The analogy is of course not complete. Quantum objects obey the Schrödinger equation, that is linear, whereas synchronization phenomena occur in nonlinear systems. Therefore, it should be perhaps more correct to say that such correlation arrives from resonances at the microscopic level. Another similarity of quantum correlations with their classical counterparts is that both are connected to phase transitions and the emergence of new properties. As for the brain, phase transitions between synchronized and desynchronized states in networks are commonplace, and they may even lead to pathological states in epilepsy and Parkinson disease, or to altered states of consciousness. Such phase transitions are important because they constitute the basis for “emergence phenomena”, a class to which consciousness seems to belong. Apart from neuroscience and many other areas of biology, emergency has been also traditionally studied by condense matter physics, in the context of large systems containing interacting units. Indeed, the title of the seminal paper by Anderson “More is different” [57], succinctly summarize what emergency is: new, unexpected properties that appear in large systems of interacting particles. Of great importance in this context, some of these phenomena are quantum in nature, as is the case of superconductivity and superfluidity. Indeed, they constitute “macroscopic quantum phenomena”, leading to emergent properties and phase transitions. For years the possibility of such macroscopic quantum phenomena in the brain were excluded, because they normally appear at very low temperatures, far from the physiologically relevant temperatures at which an organism function normally.

However, the last decade has witnessed the development of a the emerging field of quantum biology [58,59], and several unexpected quantum effects have been discovered in soft, biological tissues at room temperature, at times having crucial physiological implications, such as the discovery of quantum coherence in the photosynthesis process [60,61,62]. Other examples of relevancy of quantum effects in biology include enzyme catalysis, olfaction, the avian compass, and DNA replication [63,64]. Such advances demonstrate that decoherence may fail to destroy quantum correlations in biological tissue at physiological temperature (in the “warm, wet, and messy” biological tissue [64]), and it actually seems that at least in some cases molecular architecture and function has been designed in such a way as to avoid the deleterious effects of noise, in order to preserve quantum coherence and in a way as to benefit living organisms [65]. As well, it has been speculated that evolution may have taken advantage of such processes to maintain ordered states in crucial biological functions that could be otherwise difficult to preserve. Some authors have even gone further and suggest that nontrivial quantum effects are actually necessary for life and in particular for neural function [66].

However, how can decoherence be avoided in living organisms? In principle, due to collisions and other interactions with molecules at physiological temperatures, the delocalization of macromolecules should be short lived (from a few hundred femtoseconds to nanoseconds) and extend to short distances (few nanometers) as compared to biologically relevant scales [67]. However, some authors have proposed that there could be shortcuts within biological tissues to overcome this problem. One possibility is the existence of subspaces (such as hydrophobic pockets) within which molecules are protected from decoherence [68]. Other authors have proposed quantum error correction for the implementation of robust quantum computation [69] a method that in principle could have been implemented by living systems. The local cooling of molecular complexes could also be conceived as an effective method to avoid decoherence [70]. Another possibility is that of a dynamic entanglement that is resistant to decoherence, achieved in non-equilibrium quantum systems driven by oscillatory motion [71].

To illustrate this point, consider photosynthesis, a paradigmatic system of study in the emerging field of quantum biology [64], that sheds light on the importance of non-trivial quantum effects in living matter. In the photochemical reactions occurring during photosynthesis within plants and algae chloroplasts, light quanta is absorbed by chlorophyll, and by a chain of chemical reactions involving electron transport, solar energy is stored in the NADPH and ATP molecules, for subsequent use in metabolism [72]. Chlorophyll and other similar molecules used in photosynthesis by diverse organisms are chromophores (literally a “carrier of color”), molecules that constitute pigments in that they absorb light in certain wavelenghts and are transparent in others [73]. In the case of chlorophyll, the molecule is transparent in the wavelength corresponding to green light. One end of the chlorophyll molecule is a flat, antenna-like structure with a magnesium atom at its center. When a quantum of light hits the magnesium atom, an electron is emitted, carrying with it the energy delivered by the photon. After this initial stage, the excited electron has to be transferred to a reaction center, where charge separation occurs and free energy can be stocked within more stable molecules for subsequent use. These first stages in photosynthesis, including the transfer of energy to the reaction center, are very efficient. This is difficult to explain if it is assumed that excited electrons classically diffuse across chromophores to reach the reaction center. If this was the case, most energy would be lost and efficiency should be very low. Indeed, a search strategy based on classical Brownian motion is extremely inefficient and could not account to the almost 100% efficiency found in the first stages in photosynthesis [59,74].

Here is where quantum mechanics comes at rescue. Once an electron is excited by a quantum of light the lost negative charge can be considered as a positive “hole” in the magnesium atom, as is typically done in condense matter and field theory, a typical example being the electrons and holes used to describe semiconductors [75]. The ensemble of the electron and the hole can be treated quantum-mechanically as an elementary excitation, or exciton [76]. As it turns out, in photosynthetic light harvesting complexes these collective optical excitations lead to correlations of electron’s motions in different chromophores [77,78]. In other words, there is a superposition of the electronic excitations of individual chlorophyll molecules, leading to much more efficient transfer of energy from chlorophyll molecules to the reaction center. This possibility was already pointed out by pioneer work of Franck and Teller [79] but it was not corroborated experimentally until recently. Indeed, nowadays, there seems to exist strong evidence of wavelike energy transfer through quantum coherence in photosynthetic systems [64,80], but also see [81,82,83] for an opposing view). This was possible originally with femtosecond transient absorption spectroscopy [84,85,86,87,88,89] and more recently with two dimensional electronic spectroscopy ([90]). The first observations where done on the Fenna–Matthews–Olson (FMO) complex of (photosynthetic) green sulfur bacteria at cryogenic temperatures [60], but subsequent studies extended the original results to cover physiological temperatures and other organisms including plants [61,91,92].

## 5. Connectionist Approach to Quantum Consciousness

In spite of the growing evidence of quantum effects in biology, at first sight it seems improbable that quantum mechanics could play any significant role in any brain process involved in the psychological features associated with conscious awareness. Quantum effects are tiny compared to the scale of these physiological or cognitive events, and the consensus is that the mechanisms underlying brain function are well understood and can be described in terms of classical physics. Indeed, the attitude towards such proposals from the neuroscientific community has been typically negative. One crucial argument against the existence of quantum effects in the human brain is that decoherence should destroy such effects in the high temperature, noisy biological tissue that constitutes the nervous system. Indeed, macroscopic quantum phenomena such as superconductivity and superfluidity occur in a cryogenic regime. For many years, this argument has been a fundamental limitation to the notion of consciousness arising from purely quantum effects. However, as outlined in the last section, recent experimental results in the new field of quantum biology have made this argument much less stringent, as it is now clear that important physiological processes such as photosynthesis would benefit from non trivial quantum effects such as long-lived quantum coherence.

Another argument against a possible role of quantum physics in brain function is that there is no need for it as an explanatory aid: why use sophisticated and more mathematically involved ideas from quantum physics when neuroscience can be expressed in terms of very simple concepts requiring at most high school physics? Nevertheless, we should be cautious about this point: as we discuss later, some authors have suggested that consciousness could be irreducibly quantum (for reviews see in [12,93]).

Under this new light, a question can be naturally posed: at what level does consciousness appear? At first sight it seems that either consciousness is the result of the classical, chemical, and electrical interactions between neurons, and in particular is the result of the integration of information on large neuronal networks, or it arises within cellular structures inside neurons via quantum coherence.

We propose that these two perspectives actually have a lot in common and that perhaps consciousness is an emergent phenomenon at several spatio-temporal levels within the nervous system. As explained above, we propose a general connectionist approach, in which consciousness naturally arises as an emergent phenomenon in brain networks in which an equilibrium is reached between noise and correlation. In the following we very briefly examine several possible intracellular candidates to such types of networks within the realm of quantum theories of consciousness, and we put them in the context of a general connectionist theory of consciousness.

Perhaps the most important candidate for a subcellular neural correlates of consciousness are microtubules [94,95]. Microtubules are polymers of the protein called tubulin, and constitute the main part of cytoskeleton in eukaryotic and some prokaryotic cells [72]. They form, together with microtubule-associated proteins (MAP), actin, and intermediate filaments, a dense network inside those cells. Microtubules have important physiological roles: they constitute the “skeleton” of cells (contributing to its shape and structure and connecting organelles) and are crucial for molecular transport within cells, by means of kinesin motor proteins. They are also central to cell division. Microtubules are constantly created within the cytoplasm, by polymerization of tubulin dimmers (α and β tubulin proteins ).

According to one of the most outstanding theories of “quantum consciousness” in the brain, the orchestrated objective reduction theory (Orch OR), quantum coherence in networks of microtubules within neurons constitute a neural correlate of consciousness [96,97]. (We alert the reader that several aspects of Orch OR that we do not mention here, are highly speculative and controversial (see pages 79–112 in Volume 11 Issue 1 of Physics of Life Reports for a detailed discussion and contributions to this debate from several authors); we focus only on those less controversial aspects that are related with quantum coherence in microtubules networks.) Orch OR focuses on neurons, and ,in particular, in the neuronal soma (cell body) and dendrites. The reason is that microtubules within dendrites and neural soma are interrupted and of mixed polarity, and are connected to MAPs to form recursive networks that prevent them to disassemble [12,97]. On the contrary, microtubules in axons are unipolar and continuous, forming radial, regular arrangements. In cells other than neurons, microtubules are unstable, disassembling in various ways [12]. Therefore, the authors of Orch OR suggest that microtubules networks within neurons body and dendrites (contrary to those in axons and within other types of cells) could better support information processing.

According to Orch OR there are several reasons why microtubules networks are associated to consciousness [97]. The most important argument relates to the action of anesthetics. Indeed, anesthetics constitute a probe into consciousness. According to Turing, “the only thing we are sure about consciousness is that it is soluble in chloroform” [98]. Crucially, during anesthesia consciousness is suppressed, whereas neuronal activity can still be measured in the brain [94]. Furthermore, certain anesthetics that influence cognition and conscious experience seem to involve microtubules [12]. Relatedly, anesthetics are effective on any organism [99]. How can anesthetic inhibit some responses to the environment in unicellular organisms such as slime molds that do not even have a nervous system [100,101]? This cannot be explained by a general anesthetic action on synaptic effects [12,102,103]. Reletadely, complex responses in simple organisms such as the paramecium seem to be connected to the cytoskeleton. Therefore, it could be hypothesized that cognition in general and in particular consciousness (or loss of it through anesthesia effects) are linked to the cytoskeleton and in particular microtubules. They may constitute, for unicellular organisms but also for larger organism, what neurons constitute for the brain: basic information processing units.

Furthermore, the authors of Orch OR argue that, while action potentials (neuronal firing) represent a main mean of communication between neurons giving rise to synaptic potentials, it is the integration of these synaptic activity in the dendrites and cell bodies that is fundamental for neuronal information transmission. Also, the authors point out that gamma synchronization is related to somatic and dendritic integration of potentials [97]. And, as pointed out above, microtubule networks seem to be more suitable for information processing in bodies and dendrites of neurons. Therefore, they identify gamma synchronization with microtubules networks.

In the original version of Orch OR, Hameroff and Penrose explored the possibility of a quantum superposition of mechanical conformations coupled to London force electric dipoles. In later versions of the theory, they locate the quantum effects within the constituent aromatic rings (phenylalanine, tyrosine, and tryptophan) that make up the tubulin proteins [12], mediated via magnetic dipoles. In any case, they propose the existence of temporal correlation between microtubules, sustained by quantum coherence. The authors even go further and reason that a network of microtubules connected by quantum coherence can develop not only inside neurons but, via their connection with electrical and chemical synapses, they could extend across entire brain areas [97]. In this sense, it would be very difficult to distinguish a network of microtubules connected by quantum coherence or even entanglement, from a network of neurons connected by more familiar, synaptically-related connections and neural synchronization. One crucial distinction could be that if microtubules constitute a network where information is processed, as in Orch OR, the potential possibilities for the brain are enormously enhanced, because they constitute a dense network inside neurons.

However, it remains to answer for how long can quantum coherence be maintained in microtubules at physiological temperature. According to Tegmark, not for too long to be important in a neurophysiological context, because decoherence destroy quantum correlation between microtubules after approximately $10^{-12}$ s, too short a time lapse as compared with the scale of neuron firing (milliseconds, $10^{-3}$ s) [10]. However, a re-calculation due to Hagan et al., more in accordance to Orch OR theory, produced decoherence times of the order of 10 to 100 μs ($10^{-5}$ s–$10^{-4}$ s), much closer to the time scales of neural activity [104].

Going back to anesthetics, there are quantum effects that seem to be implicated in the action of general anesthetics [94,97]. These are better understood by reasoning in analogy with photosynthesis. Indeed, as we sketched above, photosynthesis energy transfer and charge separation is enhanced by quantum effects. Quantum betas are present in light-harvesting complexes in plants and bacteria [60]. Similarly, triptophan residues in tubulin proteins are also capable of quantum coherence transfer, at least according to theoretical models [105]. Craddock and co-workers point out that a possible action of anesthetics is the inhibition of quantum coherence between microtubules [106]. More specifically, the authors argued that anesthetics act on “quantum channels” (hydrophobic pathways of tryptophan rings in tubulin). Other, very recent evidence comes from spin changes in anesthetized fruit flies. Anesthetic action seems to generally disrupt electronic activity, suggesting that nuclear spin of anesthetic molecules may influence their efficiency [107].

Apart from research related with microtubules, the possibility of spin (which is an intrinsically quantum property) having an influence on conscious phenomena has been worked out by several authors. An important line of research in this sense examines quantum entanglement between atoms associated with crucial neural phenomena such as action potentials. Wu and Hu proposed a pioneer theory of spin-mediated consciousness, based on entanglement between different types of atoms within cell membranes [108]. More recently, Fisher built a theory of quantum cognition by processing information with entangled phosphorus atoms. Phosphorus is a crucial substance for organisms; it can be found, for example, within metabolically important molecules such as ATP. In particular, it is found within Posner molecules $Ca_{9}{\left(PO_{4}\right)}_{6}$ , believed to be important for bone formation [109]. According to Fisher and co-workers, entanglement of phosphorus atoms within Posner molecules is “protected” form the environment, in the sense that decoherence times are very long (of the order of hours or even days [110,111]). Although other authors estimated a much shorter decoherence time (37 min or even much shorter), it is in any case much longer than those estimated in quantum coherence in microtubules. In Fisher’s proposal, entangled Posner molecules could elicit the simultaneous firing of neurons, by a complex set of events leading to the release of neurotransmitters in the corresponding neurons. The mechanism through which entangled Posner molecules release neurotransmitters remains hypothetical, but the result would be that of temporal correlation of the activities of neurons containig entangled Posner molecules.

Finally, other studies point towards the possibility of quantum entanglement of photons in the brain (for a review of such ideas see [12]). For example, it has been hypothesized using a theoretical model, that photons can be used for communication between neurons, by traveling though myelinated axons, that could serve as waveguides, in analogy with the way photons travel in optical fibers [112]. Of course, such ideas remain speculative.

Whatever the details of such quantum entanglement mechanism, the relevance for the current paper is that entangled atoms or photons within neurons can be correlated across the whole brain, effectively constituting a network of correlations, or functional network, exactly as those described in neuronal network theories of consciousness.

Summarizing, we identify two types of quantum correlations in subcellular neurons that could in principle be related to cognition and conscious experience: The first is quantum coherence between adjacent structures, such as microtubules or other cytoskeleton structures, leading to coordinated activity within neurons. The second is whole brain (or at least macroscopic) correlations between entangled atoms within molecules, in turn leading to a cascade of events synchronizing neural activity. To our knowledge, there is little if no experimental evidence that any of these processes are directly related to cognition or consciousness, and for now they rely on hypothetical thinking and indirect evidence. One such indirect evidence comes from the field of quantum biology, and in particular for photosynthetic light harvesting complexes, where both quantum coherence and entanglement has been demonstrated.

An important clue to understand the possible importance of quantum effects in cognition comes from the effect of noise in biological tissue, and in particular the effect of noise on information processing. As we explained above, conscious states tend to appear at intermediate levels of noise and correlation in functional networks [38]. At first sight, it seems paradoxical that consciousness is achieved at intermediate levels of noise. Indeed, normally, noise is considered as a hindrance for information-processing systems and devices. However, there are instances where noise can be beneficial in biological systems, specially in information processing in the nervous system, where it can even enhance neural processing [113,114,115]. A paradigmatic example is stochastic resonance, where a sub-threshold input can be detected at appropriate levels of noise, and not at lower levels [116]. Strikingly, in the phenomenon of psychophysical stochastic resonance, an image embedded in noise is not visible until sufficient noise is added [117]. There is also experimental evidence that stochastic resonance enhance transport in biological systems [118,119].

Interesting, converging ideas appear in apparently unrelated studies of transport in quantum systems. Indeed, one question that puzzled researchers for decades (starting with Schrödinger in his masterpiece “What is life?” [120]) is how can certain biological processes be possible in spite of molecular noise. New developments in quantum biology points toward the possibility that an effect similar to stochastic resonance, that is, maximal efficiency of certain processes at intermediate levels of noise, also operates at the quantum level of description, and can be applied to important physiological processes related to charge and energy transport, such as photosynthesis and respiration [121,122,123].

A particularly striking example comes from models of photosynthetic complexes in bacteria: the Fenna–Matthews–Olsen (FMO) complex already mentioned above, that has been thoroughly studied by means of X-ray crystallography and spectroscopy [61,92].

This system is so well known that a model can be constructed with a single free parameter: temperature [74]. As it turns out, the passage of excitons from chlorophyll molecules to the reaction center can be modeled as a “quantum Brownian motion”, explaining the large efficiency of the transport processes [56].

The efficiency is, however, temperature-dependent [74]. At very low temperatures, interference of the different possible paths taken by the exciton are mainly destructive, leading to a localization of the exciton after a few molecular steps. At higher temperatures, the environment induces a stronger decoherence of the exciton paths, decreasing the destructive interference and therefore increasing the distance achieved by the exciton, that can eventually arrive to the reaction center. At even higher temperatures, decoherence is so strong that that the exciton essentially does not move. Maximal efficiency was reached at approximately 290 K, and maintained for several degrees Kelvin in both directions [74]. This is the range of temperatures of the water within which these bacteria live. Within this range, efficiency of transport was very close to 100%. We emphasize that the only parameter changed in the model was temperature, which is a measure of molecular disorder and can be easily interpreted as “noise”. In other words, at large and low levels of “molecular noise” the exciton does not move very far, whereas at intermediate levels maximal efficiency is achieved.

Interestingly, this kind of effect should be present in any kind of partially coherent quantum transport process in a disordered system, not only in photosynthesis and was baptized by the authors of the model as Environmentally Assisted Quantum Transport (ENAQT). Furthermore, the authors suggest a “quantum Goldilock effect”, in that natural selection drives certain biological systems into a level of quantum coherence at which optimal efficiency is achieved. This speculation is sustained by the observation of a convergence of temporal scales in photosynthesis [74,124].

**Figure 2:**
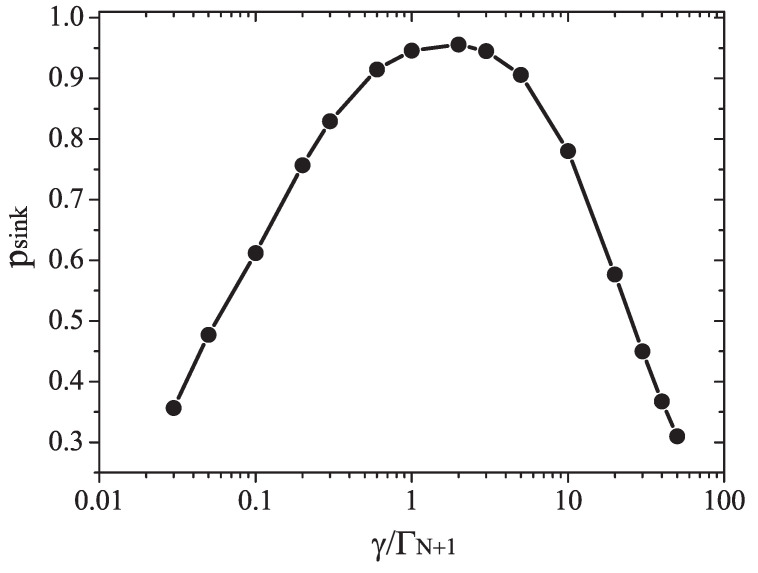
Excitation transfer in a fully connected network modeling a light harvesting complex. The probability of excitation transfer to the reaction center, or “sink” ($p_{sink}$) is a function of the noise in the environment. Noise is quantified by the local dissipation rate $\Gamma_{N+1}$, the transfer of energy from the exciton to individual nodes in the network, and a pure dephasing noise rate γ, randomizing the phase of the exciton and therefore destroying quantum coherence. Maximal exciton transfer efficiency is achieved at intermediate levels of noise and coherence. The network is constituted by N=5 sites. Reproduced from [121], with the permission of AIP Publishing.

A concrete example of this kind of effect in shown in Figure 2, which shows the influence of noise on the transfer rate of excitons in a model of light harvesting complex. An optimal performance in excitonic transfer is achieved at an intermediate level of noise and quantum coherence. Too much noise or too little are detrimental for conduction [125,126].

We speculate that the quantum Goldilock effect is the analogous, at the quantum level, to the fine-tuning between noise and coherence resulting in conscious states in the brain, observed during epileptogenic activity and sleep and described above [38]. During epileptic seizures and deep stages of sleep, brain functionality diminishes as compared with normal states, and the information in brain networks also diminishes. We may say that the corresponding brain activity is not optimal, and the brain is less efficient at information processing. Comparing Figure 1 and Figure 2 may be illustrative in this sense. In both figures efficiency is achieved at intermediate levels of noise and coherence. Huelga and Plenio suggested that the optimal efficiency in transport is achieved at an intermediate level, located between the classical regime, where dephasing noise dominates, and the quantum one, dominated by coherence [125]. Based on these observations, it is inviting to conjecture that a general principle, the self-organization of highly correlated networks, underlies the emergence of conscious phenomena.

We emphasize the hypothetical character of this idea, especially regarding the possibility of “quantum consciousness” underlying conscious phenomena (whereas its applicability to large neuronal networks seems to be much less doubtful). In this sense, it is important to note that the evidence reviewed above in favor of quantum theories of consciousness is indirect and it is not clear yet if non-trivial quantum effects are fundamental for brain function or cognition (although it is clear that quantum effects influence brain activity at the molecular level, as we mentioned above [9]). The subject is too new, and there is not enough experimental evidence in one sense or another. Even some basic results in quantum biology are under debate [83]. However, on the basis of what theories of quantum consciousness propose and the existing indirect experimental evidence shows, we have identified unifying principles that seem to underlie theories of consciousness independently of the scale. Such principles are actually not new. Increased complexity is a central theme in the science of emergence, as already recognized by Anderson in his seminal paper cited above [57], where he highlighted the importance of broken symmetries for the emergence of higher levels of complexity. Interestingly, in that work Anderson foresaw the relevance of oscillatory patterns in biological systems, speculating about the possibility of a new type of broken symmetry, that of “ordering (regularity or periodicity) in the time dimension”, that added up to the already known broken symmetries of physics and chemistry. The importance of oscillations and synchrony for the nervous system is nowadays out of question. In line with Anderson’s program, we propose that such temporal symmetries are broken in order for conscious phenomena to appear. Indeed, as pointed out above, a brain that is too regular in the temporal dimension cannot be fully functional, as is the case during epileptic seizures, dominated by quasi-periodic neural activity. Is it possible to characterize the direction of such phase transitions, leading to increase complexity and perhaps the emergence of consciousness? At the level of classical, large-scale neuronal networks, we already mentioned that the emergence of consciousness could be accompanied by the increase of energy dissipation and a decrease in free energy [35], in line with the free-energy principle proposed by Friston [46]. Do similar principles operate at the quantum level? There is indirect evidence that this could be the case. Indeed, many of the processes analyzed in quantum biology are types of quantum transport processes for which dissipation is crucial. Moreover, it has been proposed that entanglement could be sustained and decoherence avoided in nonequilibrium open quantum systems, as is the case for biological systems [71]. This opens the possibility of quantum effects to be preserved in spite of the high temperatures and noisy environments typical of living matter. Furthermore, in a recent revision of the quantum aspects of photosynthetic light harvesting, it has been proposed that dissipation is used, and not avoided, in order to direct the transport of energy in photosynthetic complexes [83]. This is achieved by the interaction between the excitons and the thermal bath. The bath is not specifically designed to avoid decoherence, but rather to facilitate energy flow. In this sense, photosynthetic function is more closely driven by thermodynamic parameters than it was assumed in previous accounts of quantum biology. In the words of the authors: “The basic physics behind thermalization is used to impose direction. This simple concept, mastered by nature over all relevant time and spatial dimensions, is truly a marvel of biology.”

## 6. Conclusions

The main question we have tried to address can be summarized as follows. What is the size of the smallest illuminated spots, the building blocks in the Cartesian theater of consciousness mentioned above? We have identified fundamental principles common to theories of consciousness, both classical and quantum. We have presented plausible arguments in favor of a multiscale connectionist approach to consciousness, with quantum effects playing a possible role in enhancing the generation of conscious phenomena. The experimental evidence in favor of such idea is not too clear, but there are certain puzzling questions about the mechanism of action of general anesthesia that may require the incorporation of quantum physics. Moreover, the last years have witnessed the development of the new field of quantum biology. Quantum coherence and entanglement seem to play a role in several physiological processes. Under this perspective, it is instructive to analyze the problem of the emergence of consciousness under a generalized connectionist point of view, where networks associated to conscious experience appear at different scales, including subcellular, and can be affected or enhanced by quantum correlations. We identify quantum coherence and entanglement as possible sources of temporal correlation, in analogy as neural synchronization supposedly underlies large neuronal network information integration. We also identify the quantum Goldilock effect to be analogue to the fine tuning of parameters observed in global neuronal networks during the emergency of consciousness. In both cases, it is the interplay between noise and correlation in the system that leads to an increase in system’s efficiency. We speculate that is this constitute a general principle underlying the emergence of conscious awareness.

This principle is most clearly understood within a thermodynamic context. We propose that the emergence of consciousness results from minimization of free energy and the corresponding increase of energy dissipation as is the case for other emergent phenomena in nonequilibrium systems. This claim is supported by neurophysiological evidence in large-scale neuronal networks. At the quantum level, recent accounts in quantum biology also point in this direction, emphasizing that biological systems are open, nonequilibrium systems, where quantum behavior can be preserved at physiological temperatures and where dissipation is not a hindrance but rather helps the process of energy transport within molecular complexes.

The concept that consciousness is a multiscale phenomenon is still a speculative idea and further research is needed in order to prove or disprove it. In particular, direct evidence is needed in support of the idea that nontrivial quantum effects can influence or produce conscious phenomena. One difficulty in this sense is to separate possible direct quantum effects (for example, in microtubules within neurons) from the cascade of neural activity that they may generate. Furthermore, most of the research in quantum consciousness relates with the action of anesthetics, which is associated to the lack or at least the partial suppression of consciousness. Can it be that other types of conscious phenomena related to cognition and perception are affected by the same physical effects? Can entanglement or quantum coherence be observed at macroscopic scales, such as between different brain areas? Detailed experimental evaluation of these possibilities is still needed.

Finally, the philosophical consequences of this idea require further exploration. Are multiscale emergent phenomena common in nature? As discussed above, life could be an example of multiscale phenomenon, if we decide that viruses as well as multicellular organisms are both alive. However, the limit between living and non-living matter is difficult to outline, at least in the philosophical sense. Similar problems appear when analyzing other emergent structures such of complex societies or the weather. Future work on several disciplines ranging from the natural and social sciences to philosophy and epistemology would be needed in order to respond to this crucial question.
